# T-Cell Activation and Early Gene Response in Dogs

**DOI:** 10.1371/journal.pone.0121169

**Published:** 2015-03-24

**Authors:** Sally-Anne Mortlock, Jerry Wei, Peter Williamson

**Affiliations:** 1 Faculty of Veterinary Science, The University of Sydney, NSW 2006, Australia; 2 Sydney Medical School, The University of Sydney, NSW 2006, Australia; Queen's University Belfast, UNITED KINGDOM

## Abstract

T-cells play a crucial role in canine immunoregulation and defence against invading pathogens. Proliferation is fundamental to T-cell differentiation, homeostasis and immune response. Initiation of proliferation following receptor mediated stimuli requires a temporally programmed gene response that can be identified as immediate-early, mid- and late phases. The immediate-early response genes in T-cell activation engage the cell cycle machinery and promote subsequent gene activation events. Genes involved in this immediate-early response in dogs are yet to be identified. The present study was undertaken to characterise the early T-cell gene response in dogs to improve understanding of the genetic mechanisms regulating immune function. Gene expression profiles were characterised using canine gene expression microarrays and quantitative reverse transcription PCR (qRT-PCR), and paired samples from eleven dogs. Significant functional annotation clusters were identified following stimulation with phytohemagluttinin (PHA) (5μg/ml), including the Toll-like receptor signaling pathway and phosphorylation pathways. Using strict statistical criteria, 13 individual genes were found to be differentially expressed, nine of which have ontologies that relate to proliferation and cell cycle control. These included, prostaglandin-endoperoxide synthase 2 (*PTGS2/COX2*), early growth response 1 (*EGR1*), growth arrest and DNA damage-inducible gene (*GADD45B*), phorbol-12-myristate-13-acetate-induced protein 1 (*PMAIP1*), V-FOS FBJ murine osteosarcoma viral oncogene homolog (*FOS*), early growth response 2 (*EGR2*), hemogen (*HEMGN*), polo-like kinase 2 (*PLK2*) and polo-like kinase 3 (*PLK3*). Differential gene expression was re-examined using qRT-PCR, which confirmed that *EGR1*, *EGR2*, *PMAIP1*, *PTGS2*, *FOS* and *GADD45B* were significantly upregulated in stimulated cells and *ALAS2* downregulated. *PTGS2* and *EGR1* showed the highest levels of response in these dogs. Both of these genes are involved in cell cycle regulation. This study provides a comprehensive analysis of the early T-cell gene response to activation in dogs.

## Introduction

The immune system is responsible for neutralizing invading pathogens through activation of a complex protective mechanisms relying on the concerted action of inflammatory and immune cells, regulatory mediators (cytokines), antibodies and complement molecules. T-lymphocytes are a key sub-population of immune cells that are vital for immunoregulation and cytotoxic effector responses. Proliferative responses are a fundamental feature of T-cell biology, and in response to receptor activation, they undergo a high rate of proliferation during development and in response to antigen induced activation. Control of the proliferative response is mediated by temporally programmed gene expression that can be identified as immediate-early, mid- and late phases [1–3]. Amongst other things, the early response genes lead to engagement of the cell cycle machinery, and regulate downstream molecular and cellular events [2]. These events are key determinants of an adaptive immune response and will dictate cell-mediated immunoregulatory and homeostatic outcomes.

Activation of T-cells in humans and mice, using either a combination of receptor agonists or mitogens, leads to intracellular phosphorylation events and induction of signalling cascades [4–6]. These events activate and regulate multiple pathways including those that involve mitogen-associated protein kinase (*MAPK*), extracellular signal-regulated kinase (*ERK*), c-Jun N-terminal kinases (*JNK*), nuclear factor-κB (*NF-κB*) and nuclear factor of activated T-cells (*NFAT*) [3–5, 7]. A study investigating gene expression profiles of human lymphocytes revealed groups of functionally similar genes showing distinct expression patterns following activation over a 48hr period [2]. The most immediate changes in expression were seen in transcription factors (*EGR1*, *EGR2*, *NF-κB p50*), followed by cytokines, adhesion molecules and genes involved in signalling pathways (RAB13, TNF receptor associated factor) [2]. After a longer period of activation genes involved in DNA replication (DNA Topoisomerase II), cellular proliferation, metabolic enzymes and structural proteins (ribonuclease) are induced [2]. Similar results have been shown in mice, with significant changes in expression of functional groups affecting transcription, signal transduction, cell division and cell surface receptors [7].

Molecular events during T-cell activation are poorly understood in dogs, with no reported studies investigating the early response of genes to lymphocyte activation on a genome-wide scale. A small number of studies have instead focused on changes in expression of individual genes in diseased states [8]. The present study was undertaken to characterise T-cell proliferation in dogs using global expression analysis to identify and explore the variation in expression of key genes, with a specific focus on the early T-cell gene response. T-cell activation was initiated using phytohemagglutinin (PHA) a widely used mitogen which binds primarily to T-cells subsequently stimulating rapid and robust T-cell proliferation [9, 10]. PHA has been successfully used in multiple species to stimulate T-cell proliferation including dogs [6, 11, 12] and is known to closely reflect the gene response of T-cells activated by TCR [13, 14].

## Methods

### Blood collection and cell stimulation

A total of eleven dogs (*Canis lupus familiaris*) were selected for inclusion in the gene expression analysis, both sexes were represented with an age range from eight months to seven years (Table 1). Dogs used in the study were all privately owned pets and were volunteered with informed consent; they remained under the care of their owners and were not housed for the purposes of this study, or held for any duration of time. Blood was collected into EDTA coated vacutainers, and the leukocyte fraction was isolated by gradient centrifugation. A registered veterinarian conducted the blood draw procedure in the presence of the owners with minimal discomfort to the animal. Cells were divided into two aliquots and each was resuspended in 1 ml Dulbecco’s Modified Eagle Medium media. Both aliquots from each dog were incubated for 4 hours, one in the presence of 5μg/ml of PHA, a mitogen for T-cell activation (referred to as the stimulated sample), the other aliquot served as the non-stimulated control sample. After incubation the cells were collected by centrifugation, and then lysed in RLT buffer (QIAmp; Qiagen Aust). Once lysed samples were immediately frozen until further use (within 48hr).

**Table 1:** Details of dogs used in the study and their corresponding sample name and treatments.

Dog No.	Dog age	Dog Sex	Sample	Treatment
1	4yrs	M	SM1	Unstimulated
			SM1P	Stimulated
2	4yrs	F	SM3	Unstimulated
			SM3P	Stimulated
3	15 months	M	SM4P	Stimulated
4	2.5yrs	F	SM9	Unstimulated
			SM9P	Stimulated
5	4yrs	F	SM13	Unstimulated
			SM13P	Stimulated
6	2yrs	F	SM14	Unstimulated
			SM14P	Stimulated
7	7yrs	F	SM16	Unstimulated
			SM16P	Stimulated
8	7yrs	M	SM17	Unstimulated
			SM17P	Stimulated
9	8 months	M	SM5	Unstimulated
			SM5P	Stimulated
10	15 months	F	SM6	Unstimulated
			SM6P	Stimulated
11	1yr	F	SM10	Unstimulated
			SM10P	Stimulated

### RNA isolation

Total RNA was isolated from samples using a modified extraction procedure and micro-spin columns (QIAmp; Qiagen, Melbourne, Vic). The lysate was pipetted directly into a QIAshredder spin column following incubation in a 37°C water bath until completely thawed and salts dissolved. 70% ethanol was added to the homogenised lysate before it was transferred into a QIAamp spin column and washed using a high-salt buffering system allowing RNA to bind to the QIAmp membrane as contaminants are removed. Purified RNA was eluted in 30 μl of RNase-free water followed by a second elution in 50 μl and stored at -80°C.

### Microarray Analysis

Frozen RNA samples from stimulated and non-stimulated cells from eleven dogs were transported to the core facility for processing (Ramaciotti Centre for Gene Function Analysis, Sydney). RNA quality and integrity was confirmed with a bioanalyzer (Agilent Technologies, CA, USA) providing an RNA integrity number for each sample, all of which were >7. RNA was then amplified and labelled before being hybridised to an Affymetrix Canine Gene 1.0 ST Array according to the manufacturer’s instructions. Array chips were read using the GeneChip Scanner 3000 (Affymetrix) and probe intensities obtained using GCOS operating software (Affymetrix). The data discussed in this publication have been deposited in NCBI’s Gene Expression Omnibus [15] and are accessible through GEO Series accession number GSE65158.

### Data Analysis

The Affymetrix Canine Gene 1.0 ST Array data from dogs one to eleven was first analysed for technical faults; one non-stimulated sample (SM4) failed to amplify and was not included. Background correction and quantile normalisation were performed on the data with probe-set intensities calculated accordingly using the Robust Multichip Average (RMA) [16] algorithm available in the Affymetrix Expression Console. Differential expression (DE) of genes between stimulated and unstimulated T-cells was defined using the limma function (linear model approach) [17] incorporated in the R package [18]. A rank fold-change was used to identify DE genes, with a cut-off of ±2-fold, corresponding to a Benjamini & Hochberg adjusted false discovery rate (FDR) of 0.05 [19]. Differentially expressed genes were then analysed for functional annotation clustering using DAVID (The Database for Annotation, Visualization and Integrated Discovery) with a group enrichment score cut-off of 1.3 (EASE = 0.05) [20]. The EASE score is the geometric mean of all the enrichment p-values of members within each annotation cluster. The group enrichment score represents the EASE score (in—log scale), ranking the clusters according to their biological significance [20]. Cluster analysis of expression data from DE genes and the gene set corresponding to the Toll-like-receptor signaling pathway were performed using Heatmap, an integrated function implemented in R, with additional annotations sourced from the program Heatplus [21].

### Quantitative Reverse Transcription PCR

Additional quantitative analysis for individual genes was performed using qRT-PCR. Oligonucleotide primers were designed for ten differentially expressed genes, chosen for their roles in cell cycle regulation and proliferation, namely *EGR1*, *EGR2*, *PMAIP1*, *PLK2*, *PLK3*, *PTGS2*, *RGS1*, *FOS*, *GADD45B* and *ALAS2*, and two reference genes *SDHA* and *GAPDH* using Primer3 software (http://bioinfo.ut.ee/primer3-0.4.0/), and then synthesised using a commercial source (Invitrogen. Melbourne, VIC, Australia) (Table 2). First strand cDNA synthesis was performed using RNA from both stimulated and unstimulated samples from six of the eleven dogs by the GoScript Reverse Transcriptase System according to manufactures instructions (Promega, Australia). RNA from the additional five dogs was unavailable for use in this experiment. Quantitative PCR was performed in duplicate under the following conditions: 20 μl reaction containing 2 μl cDNA transcript, 8 μl QuantiFast SYBR Green PCR Master Mix (Qiagen, Victoria, Australia) and 8 pmol of each primer, using a Rotor Gene 6000 system (Corbett Research, NSW, Australia). The samples were denatured at 95°C for 5 mins followed by a 35 cycle PCR run of 94°C for 15 secs, 60°C for 20 secs and 72°C for 40 secs followed by a melt curve analysis. Calculation of the relative levels of expression, normalised to the reference genes, between stimulated and unstimulated samples and statistical analysis was determined using the software REST (Qiagen, Australia).

**Table 2:** Sequence of primers used in qRT-PCR reactions.

Primer Set	Forward Primer (5'->3')	Reverse Primer (5'->3')
PTGS2	ATGGGTGTGAAAGGCAAGAA	TGATGGGTAAAGTGCTGGGC
EGR1	GCTGGAGGAGATGATGCTGCT	TGTCGGGAAAAGACTCTGCGG
FOS	GGAACAGGAGACAGACCAAC	TAGGGAAGCCACGGACATC
GADD45B	CTGGTCACGAACCCTCACAC	TCAACAGGCTCTGATGCTGG
EGR2	GCCGTAGACAAAATCCCAGT	CCAAGGACCTCTTCTCTCCA
RGS1	ATTGAGTTCTGGCTGGCTTG	CGTAGGGGTTGGTGCTTTA
ALAS2	GGAGCGTGATGGAGTTATGC	GATTCTAAAGCCCCAGAGAGC
PMAIP1	CGAAGAGCTCGAAGTGGAGT	CTGAGCGGAAGAGTTTGGAT
PLK2	TCCAGCCACCTACCACTACA	TGTCTTCAAGGCATTCACTG
PLK3	TCTGGTATGGGTCAGCAAGTGG	GCACGGTCTTTCTGTTGGC
SDHA	TGTCACCAAGGAGCCAATC	ACCAAGTCCAAGAGCGAGTT
GAPDH	GCCAAAAGGGTCATCATCTC	GGGGCCATCCACAGTCTTCT

### Ethics Statement

This study was approved by The University of Sydney Animal Ethics Committee, under protocol number 444.

## Results

### Microarray analysis of T-cell response to stimulation

Second generation canine gene expression microarrays have proven to be an effective way to investigate transcriptome level responses in T-cells. In this study PHA stimulation was used for a relatively short time-frame to provide for the identification of the most immediate events, and the most responsive genes. When stimulated and unstimulated cells were compared, a total of 2914 probe IDs (representing 1302 annotated genes) were identified as differentially expressed. Of these 1302 annotated genes 665 were upregulated in stimulated cells and 637 downregulated. Applying a 2 fold-change cut-off, a total of 12 annotated genes remain (Table 3). Two genes were downregulated in stimulated T-cells relative to unstimulated cells, hemogen (*HEMGN*) and delta-aminolevulinate synthase 2 (*ALAS2*). Ten genes were upregulated in stimulated T-cells including, prostaglandin-endoperoxide synthase 2 (*PTGS2/COX2*), early growth response 1 (*EGR1*), LOC100684961, growth arrest and DNA damage-inducible gene (*GADD45B*), phorbol-12-myristate-13-acetate-induced protein 1 (*PMAIP1*), V-FOS FBJ murine osteosarcoma viral oncogene homolog (*FOS*), early growth response 2 (*EGR2*), regulator of G protein signaling 1 (*RGS1*), OTU domain-containing protein 1 (*OTUD1*) and polo-like kinase 2 (*PLK2*). Although the fold change of polo-like kinase 3 (*PLK3*) fell outside the 2 fold-change cut-off, it was included in investigations due to its role in cell division and significant P-value. The identification of these genes provides a good indication of the nature of the immediate response, and the level of response in dogs. The functional annotation clustering analysis revealed that within the significantly higher DE genes in stimulated cells there were three functional annotation clusters that had enrichment scores of more than 1.3 and were considered biologically significant. Of these one is associated with cellular structures such as organelle lumen, another receptor signaling pathways and the third phosphorylation (enrichment scores 1.5, 1.43 and 1.33 respectively) (Table 4). In contrast no functional annotation clusters with a score above 1.3 were identified in significantly lower DE genes in stimulated cells.

**Table 3:** Top differentially expressed genes between stimulated and unstimulated T-cells in dogs from the microarray analysis.

**Gene Symbol**	**Gene Name**	**Fold Change**	**P-value**
PTGS2	PROSTAGLANDIN-ENDOPEROXIDE SYNTHASE 2	5.86	<0.001
EGR1	EARLY GROWTH RESPONSE 1	5.48	<0.001
LOC100684961	LOC100684961	3.29	<0.001
HEMGN	HEMOGEN	-2.94	0.01
GADD45B	GROWTH ARREST AND DNA DAMAGE-INDUCIBLE GENE	2.63	<0.001
PMAIP1	PHORBOL-12-MYRISTATE-13-ACETATE-INDUCED PROTEIN 1	2.57	<0.001
FOS	V-FOS FBJ MURINE OSTEOSARCOMA VIRAL ONCOGENE HOMOLOG	2.50	<0.001
EGR2	EARLY GROWTH RESPONSE 2	2.37	<0.001
RGS1	REGULATOR OF G PROTEIN SIGNALING 1	2.11	<0.001
ALAS2	DELTA-AMINOLEVULINATE SYNTHASE 2	-2.10	<0.001
OTUD1	OTU DOMAIN-CONTAINING PROTEIN 1	2.03	<0.001
PLK2	POLO-LIKE KINASE 2	2.01	<0.001
PLK3	POLO-LIKE KINASE 3	0.90	<0.001

**Table 4:** Genes belonging to significantly upregulated functional annotation clusters in stimulated T-cells.

**Functional Annotation Cluster**	**Gene Name**
Organelle Lumen	CYTOCHROME C, SOMATIC
	SURVIVAL MOTOR NEURON
	ACIDIC (LEUCINE-RICH) NUCLEAR PHOSPHOPROTEIN 32 FAMILY, MEMBER A
	DIHYDROLIPOAMIDE DEHYDROGENASE
Toll-like Receptor Signaling Pathway	MITOGEN-ACTIVATED PROTEIN KINASE 8
	TRANSMEMBRANE EMP24 PROTEIN TRANSPORT DOMAIN CONTAINING 7
	INTERLEUKIN 1, BETA
	FBJ MURINE OSTEOSARCOMA VIRAL ONCOGENE HOMOLOG
	JUN PROTO-ONCOGENE
	INTERLEUKIN 8
	TUMOR NECROSIS FACTOR
	LIPOPOLYSACCHARIDE BINDING PROTEIN
	INTERFERON ALPHA-1/2-LIKE
NOD-like Receptor Signaling Pathway	INTERLEUKIN 1, BETA
	INTERLEUKIN 8
	MITOGEN-ACTIVATED PROTEIN KINASE 8
	TUMOR NECROSIS FACTOR
	TUMOR NECROSIS FACTOR, ALPHA-INDUCED PROTEIN 3
RIG-I-like Receptor Signaling Pathway	MITOGEN-ACTIVATED PROTEIN KINASE 8
	TUMOR NECROSIS FACTOR
	INTERLEUKIN 8
	INTERFERON ALPHA-1/2-LIKE
Phosphorylation	INTERLEUKIN 5
	BCL2-ASSOCIATED X PROTEIN
	SUPPRESSOR OF CYTOKINE SIGNALING 3
	INTERLEUKIN 1, BETA

### Hierarchical cluster analysis

**Fig 1:**
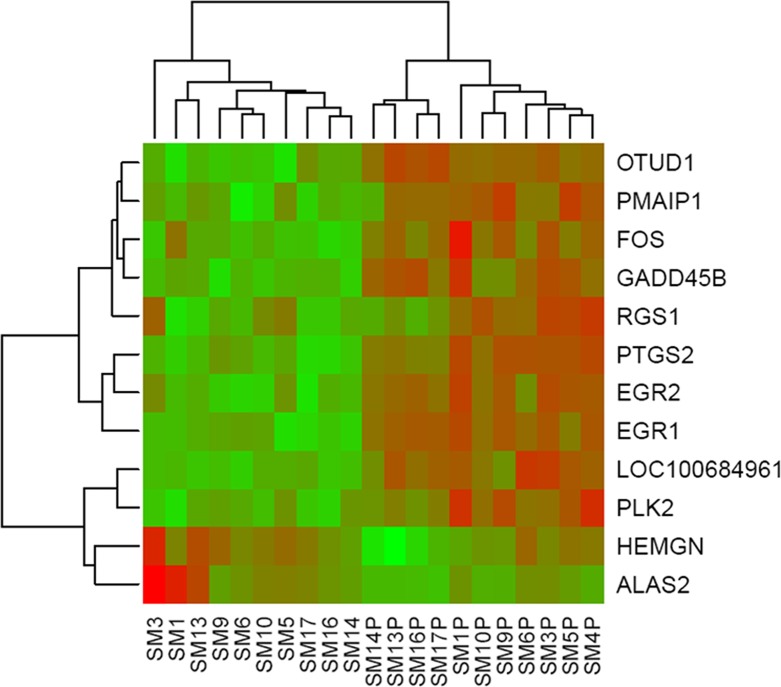
Hierarchical clustering and heat map of 12 significantly differentially expressed genes comparing stimulated and unstimulated T-cells. Expression levels are color-coded with shades of red, green and black corresponding to the degree of increase, decrease or no change in gene expression, respectively.

**Fig 2:**
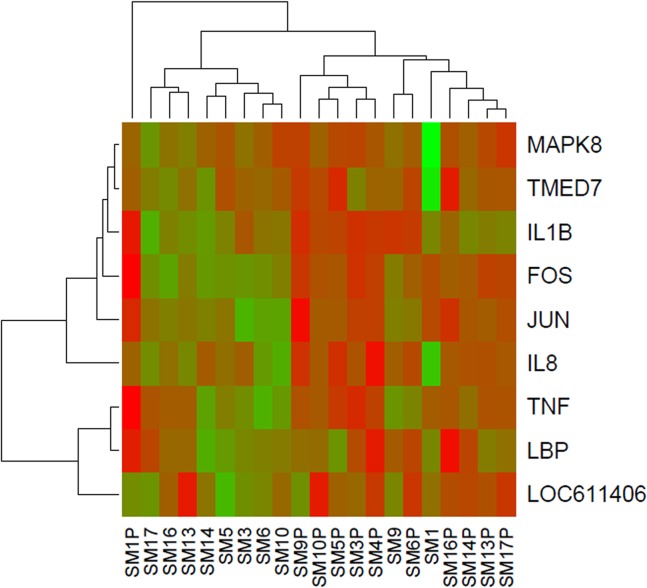
Hierarchical clustering and heat map of expression levels of genes involved in TLR signally pathways. Expression levels are colour coded with shades of red, green and black corresponding to an increase, decrease and no change in gene expression, respectively.

Hierarchical cluster analysis is a useful method for investigating consistent and robust expression profiles between treatment groups. Variation in the gene expression patterns is highlighted by hierarchical cluster analysis of DE genes. There was tight clustering of expression profiles from DE genes between stimulated and non-stimulated cells (Fig. 1). The expression profile of nine genes, representative of 76 genes in the TLR signaling pathway, resulted in a significant enrichment score in the functional annotation cluster analysis. Hierarchical cluster analysis of these upregulated genes shows distinct clusters between stimulated and non-stimulated cells with the exception of SM1 and SM9 which cluster with stimulated cells (Fig. 2).

### Quantitative Reverse Transcription PCR analysis of T-cell response to stimulation

**Fig 3:**
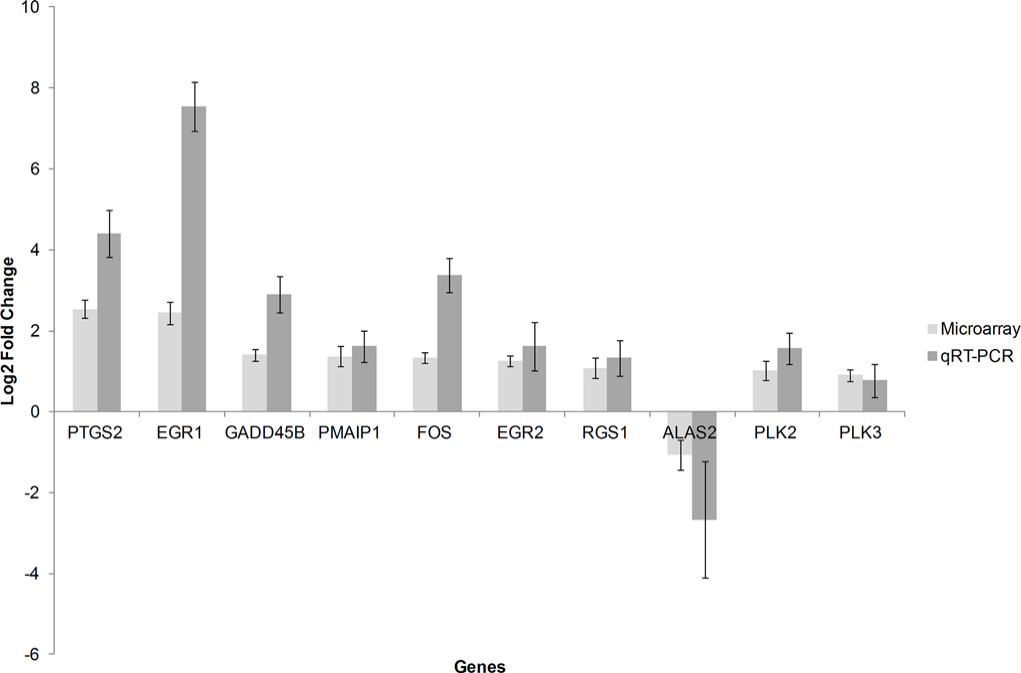
Comparison of differentially expressed genes between stimulated and unstimulated T-cells using microarray and qRT-PCR analysis (P-values are summarised in Tables 3 and 5).

Six of the ten genes were significantly upregulated in stimulated T-cells when compared to unstimulated T-cells; these include *EGR1*, *EGR2*, *PMAIP1*, *PTGS2*, *FOS* and *GADD45B* (Table 5). *ALAS2* was significantly downregulated in stimulated T-cells. *EGR1* and *PTGS2* had the greatest change in expression with *EGR1* upregulated in stimulated cells by 7.5-fold and *PTGS2* by 4.4-fold. While greater differences were observed across nine of the ten genes in the qRT-PCR when compared to those from the microarray, the patterns seen across the genes were consistent with that of the microarray (Fig. 3).

**Table 5:** Analysis of differentially expressed genes between stimulated and unstimulated T-cells using qRT-PCR.

Gene Symbol	Gene Name	Fold Change (Log2)	P-Value
EGR1	EARLY GROWTH RESPONSE 1	7.54	0.001
PTGS2	PROSTAGLANDIN-ENDOPEROXIDE SYNTHASE 2	4.40	0.001
FOS	V-FOS FBJ MURINE OSTEOSARCOMA VIRAL ONCOGENE HOMOLOG	3.39	0.001
GADD45B	GROWTH ARREST AND DNA DAMAGE-INDUCIBLE GENE	2.91	0.001
EGR2	EARLY GROWTH RESPONSE 2	1.62	0.012
PMAIP1	PHORBOL-12-MYRISTATE-13-ACETATE-INDUCED PROTEIN 1	1.62	0.029
ALAS2	DELTA-AMINOLEVULINATE SYNTHASE 2	-2.67	0.047
PLK2	POLO-LIKE KINASE 2	1.57	0.081
RGS1	REGULATOR OF G PROTEIN SIGNALING 1	1.33	0.146
PLK3	POLO-LIKE KINASE 2	0.77	0.165

## Discussion

The present study characterises the immediate-early molecular T-cell stimulatory responses in a cohort of healthy dogs. Microarray and qRT-PCR analysis of gene expression following stimulation identified key genes that control activation and cellular proliferation. Prominent amongst these were *EGR1* and *PTGS2*, with an additional seven of the top DE genes associated with pathways that relate to cell proliferation and differentiation. These genes represent the most sensitive T-cell early response genes to stimulation in dogs and may be useful sensitive quantitative markers of activation.

Functional annotation clusters, identified within the most responsive genes, highlight cellular processes that may play a key role in T-cell activation and proliferation. These clusters included the NOD-like receptor signaling pathway, the RIG-I-like signaling pathway and the Toll-like receptor (TLR) signaling pathway. Similar intracellular signaling pathways have been highlighted in both humans and mice, and play a significant functional role in T-cell activation [2, 3, 7]. NOD-like receptors (NLRs) are pattern recognition receptors found in lymphocytes, dendritic cells, macrophages and other non-immune cells. They recognise pathogen-associated molecular patterns (PAMPs) and damage-associated molecular patterns (DAMPs) and play a role in the regulation of the innate immune response. Activation contributes to regulation of the apoptotic response, inflammasome assembly and the production of proinflammatory cytokines [22]. RIG-I-like receptors also induce inflammatory cytokines, particularly in response to viral infection [23], and although a role in regulation of intrinsic mRNA is intimated, the importance of this in T-cell activation is not clear.

The nine upregulated DE genes representing the TLR signaling pathway had distinct expression patterns between stimulated and non-stimulated cells. These genes play important roles in the TLR signaling pathway *FOS*, cytokines interleukin 1-beta, interleukin 8 and tumor necrosis factor, phosphorylation mitogen-activated protein kinase 8 and transmembrane emp24 protein transport domain containing 7 are responsible for transcription, cellular signalling, phosphorylation and selection of secretory cargo respectively [24–26]. This pathway is of particular interest for its role in T-cell proliferation and regulation of the cell cycle, which is detected within hours after T-cell activation. TLR proteins recognise PAMPs in structurally conserved molecules from microbes and DAMPs released from stressed cells, and as a result activate an innate immune response. Upon activation they induce production of proinflammatory cytokines and elevate expression of costimulatory molecules. They are linked to cell proliferation, cell survival, apoptosis and tissue remodelling and repair [27–30]. The expression of TLRs on CD8 T-lymphocytes and interactions with TLR adaptor molecule myeloid differentiation factor (MyD88) has been shown to facilitate rapid T-cell activation in response to invading pathogens [31]. TLR signaling pathways influence the regulation of proteins, such as D-type cyclins and cyclin dependent kinases, which play key roles in cell cycle kinetics [32]. The TLR signaling pathway has been shown to enhance cell survival and proliferation. As seen in a study looking at TLRs in CD4 T-cells in which the engagement of TLR1/2, TLR5, TLR7/8 and TLR9 alters interleukin 2 (*IL-2*) production and increases proliferation [32].

Individual genes of interest included *EGR1* and *PTGS2*, both relate to a role in cell cycle regulation and cell proliferation. One of the genes with the greatest response to stimulation in the present study was *EGR1*, with expression increasing 5.48 and 7.54-fold in stimulated cells based on results from the microarray and qRT-PCR respectively. *EGR1* is an inducible transcription factor and plays an important role in gene transcription and T-cell development [33, 34]. Previous studies have identified EGR transcription factors as genes induced by T-cell activation in both humans and mice [2, 3, 35]. *EGR1* interacts with the key transcriptional regulator, *NFAT*, and acts as a switch in the progression of the cell cycle from the resting G_0_ to primed G_1_-phase in T-lymphocytes whilst also playing an important role in the regulation of cytokine expression, including the gene for T-cell growth factor *IL2*, promoting proliferation [33, 36, 37]. Further, it has previously been linked to the development of cancer, and is involved in the proliferation and survival of cancer cells through its direct involvement in the regulation of cyclin D2 (*CCND2*), *p19* and *Fas* [38]. *EGR1* is linked to over-expression of *CCND2*, a positive regulator of the cell cycle, which may result in deregulated cell cycle progression.

*PTGS2*, also highly expressed in stimulated cells regulates prostaglandin synthesis in association with biological processes such as inflammation and proliferation. *PTGS2* has been identified in other species as an immediate-early response gene following T-cell activation and is associated with T-cell signaling pathways, regulating proliferation of T-cells, activation of transcription factors *NF-AT* and *NF-kB* and production of *IL-2*, *TNF*-α, and *IFN*-γ [39]. *PTGS2* plays an active role in the immune response, regulating production of prostaglandin E2 (*PGE2*). PGE2 promotes local vasodilatation and the attraction, as well as activation of neutrophils, macrophages and mast cells during active inflammation [40]. PGE2 also promotes immune suppression by suppressing proinflammatory cytokines [40]. High doses of *PGE2* can also suppress the production of *IL2* and in lower doses can regulate the shift from aggressive Th1 cells towards TH2 and TH17 responses [40–44]. The immunosuppressive action of *PGE2* may also contribute to the over-expression of *PTGS2* by some tumors [40, 45, 46].

Additionally, amongst the most responsive genes in this study were: *FOS*, *PMAIP1*, *PLK2/3*, *EGR2*, *GADD45B* and *HEMGN*. *FOS* is a well known transcriptional regulator that codes a leucine zipper protein that dimerises with proteins of the *JUN* family to form the activator protein-1 (AP-1) transcription factor complex [47–49]. Genes from the Jun-Fos family have previously been implicated as rapidly induced transcription factors in response to T-cell activation [3], some studies in both humans and mice however report down regulation of JunB and C-Fos after 4 hours of stimulation, which is probably a result of a delayed timeframe for collection of data [2, 7]. Over-expression of this transcription factor is associated with increased proliferation of cells and oncogenic potential. *PMAIP1* is a negative regulator of cell cycle and a mediator of p53-dependent apoptosis, expression of this gene during T-cell activation helps to ensure that cells do not multiply uncontrollably [50, 51]. Disruption of PAIMP1 has been shown to reduce DNA damage-induced apoptosis [51]. Genes involved in the negative regulation of cell cycle progression, signaling pathways, and transcription have been shown previously to be induced early following mitogenic stimulation and play a vital role in immune modulation [2, 3]. *PLK2* and *PLK3* belong to the ‘polo’ family of serine/threonine protein kinases that play a role in cell division. *PLK2* is a transcriptional target for p53 after genotoxic stress [52, 53]. *PLK3*, also known as *CNK*, has been implicated in the phosphorylation of *CDC25C*, a phosphoprotein activated in the G2 phase of cell division and essential for cell cycle progression [54]. *EGR2* is another member of the EGR transcription factor family that has been shown to become upregulated in response to T-cell activation [2, 3]. *EGR2* is a negative regulator of cell growth [55] and is involved in regulating cell survival during the positive selection of T-cells [56]. It attenuates the immune response through negative regulation of T-cell activation, [57, 58], plays a regulatory role at different stages of T-cell development [56], and is required for initiation of T-cell anergy [57]. *GADD45B* genes are highly expressed in stimulated cells, and are activated in response to DNA damage and environmental stresses. Studies in mice have shown that activation of *GADD45B* by NF-κB downregulates the pro-apoptotic JNK signaling cascade, thereby temporarily protecting cells from apoptosis [59]. *HEMGN* is downregulated in stimulated cells. It plays a role in the proliferation, differentiation and survival of hematopoietic cells [60] and is associated with the negative regulation of lymphocyte development [61]. Over-expression of *HEMGN* has been shown to promote proliferation and enhance survival through the activation of NF- κB in myeloid cells and impede lymphoid development and differentiation [60, 61].

In conclusion, this study provides a comprehensive analysis of the early T-cell gene response to activation in dogs and improves our knowledge on the genetic mechanisms behind T-cell activation. Key differentially expressed genes and biological pathways identified here may provide quantitative markers of T-cell response.

## References

[1] Crabtree GR. Contingent Genetic Regulatory Events in Lymphocyte-T Activation. Science. 1989;243(4889):355–361. 2783497 10.1126/science.2783497

[2] Ellisen LW, Palmer RE, Maki RG, Truong VB, Tamayo P, Oliner JD, et al Cascades of transcriptional induction during human lymphocyte activation. Eur J Cell Biol. 2001;80(5):321–328. 11432721 10.1078/0171-9335-00162

[3] Kelly K, Siebenlist U. Immediate-early genes induced by antigen receptor stimulation. Curr Opin Immunol. 1995;7(3):327–332. 7546396 10.1016/0952-7915(95)80106-5

[4] Smith-Garvin JE, Koretzky GA, Jordan MS. T Cell Activation Annual Review of Immunology. Annual Review of Immunology. 27 Palo Alto: Annual Reviews; 2009 p. 591–619. 19132916 10.1146/annurev.immunol.021908.132706 PMC2740335

[5] Cronin SJF, Penninger JM. From T-cell activation signals to signaling control of anti-cancer immunity. Immunological Reviews. 2007;220:151–168. 17979845 10.1111/j.1600-065X.2007.00570.x

[6] Sofuni T, Yoshida MC. Combined use of several mitogens for mitotic stimulation to human-lymphocytes. J Radiat Res. 1992;33:222–230. 1507172 10.1269/jrr.33.supplement_222

[7] Teague TK, Hildeman D, Kedl RM, Mitchell T, Rees W, Schaefer BC, et al Activation changes the spectrum but not the diversity of genes expressed by T cells. Proceedings of the National Academy of Sciences of the United States of America. 1999;96(22):12691–12696. 10535984 10.1073/pnas.96.22.12691 PMC23052

[8] Veenhof EZ, Rutten VP, van Noort R, Knol EF, Willemse T. Evaluation of T-cell activation in the duodenum of dogs with cutaneous food hypersensitivity. Am J Vet Res. 2010;71(4):441–446. 10.2460/ajvr.71.4.441 20367052

[9] Hutchins D, Steel CM. Phytohemagglutinin-induced proliferation of human lymphocytes,t—differences between neonate and adults in accessory cell requirements. Clin Exp Immunol. 1983;52(2):355–364. 6602678 PMC1535843

[10] Wang H, Daniel V, Sadeghi M, Opelz G. Differences in the Induction of Induced Human CD4(+) CD25(+) FoxP3(+) T-Regulatory Cells and CD3(+) CD8(+) CD28(-) T-Suppressor Cells Subset Phenotypes In Vitro: Comparison of Phorbol 12-Myristate 13-Acetate/Ionomycin and Phytohemagglutinin Stimulation. Transplant Proc. 2013;45(5):1822–1831. 10.1016/j.transproceed.2012.10.061 23769052

[11] Ellner JJ, Lipsky PE, Rosenthal AS. Phytohemagglutinin-induced proliferation of guinea-pig thymus-derived lymphocytes. 2. Accessory cell-function. Journal of Immunology. 1976;116(3):876–880. 1082898

[12] Helfand SC, Modiano JF, Nowell PC. Immunophysiological studies of interleukin-2 and canine lymphocytes. Vet Immunol Immunopathol. 1992;33(1–2):1–16. 1632072 10.1016/0165-2427(92)90030-t

[13] Diehn M, Alizadeh AA, Rando OJ, Liu CL, Stankunas K, Botstein D, et al Genomic expression programs and the integration of the CD28 costimulatory signal in T cell activation. Proceedings of the National Academy of Sciences of the United States of America. 2002;99(18):11796–11801. 12195013 10.1073/pnas.092284399 PMC129348

[14] Lin ZS, Fillmore GC, Urn TH, Elenitoba-Johnson KJ, Lim MS. Comparative microarray analysis of gene expression during activation of human peripheral blood T cells and leukemic Jurkat T cells. Lab Invest. 2003;83(6):765–776. 12808112 10.1097/01.lab.0000073130.58435.e5

[15] Edgar R, Domrachev M, Lash AE. Gene Expression Omnibus: NCBI gene expression and hybridization array data repository. Nucleic Acids Res. 2002;30(1):207–210. 11752295 10.1093/nar/30.1.207 PMC99122

[16] Irizarry RA, Hobbs B, Collin F, Beazer-Barclay YD, Antonellis KJ, Scherf U, et al Exploration, normalization, and summaries of high density oligonucleotide array probe level data. Biostatistics. 2003;4(2):249–264. 12925520 10.1093/biostatistics/4.2.249

[17] Smyth GK. Linear models and empirical bayes methods for assessing differential expression in microarray experiments. Statistical applications in genetics and molecular biology. 2004;3:Article3 16646809 10.2202/1544-6115.1027

[18] Sanges R, Cordero F, Calogero RA. oneChannelGUI: a graphical interface to Bioconductor tools, designed for life scientists who are not familiar with R language. Bioinformatics 2007;23:3406–3408. 17875544 10.1093/bioinformatics/btm469

[19] Benjamini Y, Hochberg Y. Controlling the False Discovery Rate—A Practical and Powerful Approach to Multiple Testing. Journal of the Royal Statistical Society Series B-Methodological. 1995;57(1):289–300.

[20] Huang DW, Sherman BT, Lempicki RA. Systematic and integrative analysis of large gene lists using DAVID bioinformatics resources. Nature Protocols. 2009;4(1):44–57. 10.1038/nprot.2008.211 19131956

[21] Ploner A. Heatplus: Heatmaps with row and/or column covariates and colored clusters. R package version 2120. 2014.

[22] Kersse K, Bertrand MJM, Lamkanfi M, Vandenabeele P. NOD-like receptors and the innate immune system: Coping with danger, damage and death. Cytokine Growth Factor Rev. 2011;22(5–6):257–276. 10.1016/j.cytogfr.2011.11.006 21996492

[23] Kawai T, Akira S. Toll-like Receptor and RIG-1-like Receptor Signaling In: Rose NR, editor. Year in Immunology 2008 Annals of the New York Academy of Sciences 11432008. p. 1–20. 10.1196/annals.1443.020 19076341

[24] Liaunardy-Jopeace A, Bryant CE, Gay NJ. The COP II adaptor protein TMED7 is required to initiate and mediate the delivery of TLR4 to the plasma membrane. Science Signaling. 2014;7(336):12. 10.1126/scisignal.2005275 PMC4685749 25074978

[25] Akdis M, Burgler S, Crameri R, Eiwegger T, Fujita H, Gomez E, et al Interleukins, from 1 to 37, and interferon-gamma: Receptors, functions, and roles in diseases. Journal of Allergy and Clinical Immunology. 2011;127(3):701–U317. 10.1016/j.jaci.2010.11.050 21377040

[26] Cargnello M, Roux PP. Activation and Function of the MAPKs and Their Substrates, the MAPK-Activated Protein Kinases. Microbiol Mol Biol Rev. 2011;75(1):50–83. 10.1128/MMBR.00031-10 21372320 PMC3063353

[27] Chang ZL. Important aspects of Toll-like receptors, ligands and their signaling pathways. Inflammation Research. 2010;59(10):791–808. 10.1007/s00011-010-0208-2 20593217

[28] Fukata M, Chen A, Klepper A, Krishnareddy S, Vamadevan AS, Thomas LS, et al Cox-2 is regulated by Toll-like receptor-4 (TLR4) signaling: Role in proliferation and apoptosis in the intestine. Gastroenterology. 2006;131(3):862–877. 16952555 10.1053/j.gastro.2006.06.017 PMC2169292

[29] Rakoff-Nahoum S, Medzhitov R. Role of Toll-like receptors in tissue repair and tumorigenesis. Biochem-Moscow. 2008;73(5):555–561. 18605980 10.1134/s0006297908050088

[30] McGettrick AF, O'Neill LAJ. Toll-like receptors: key activators of leucocytes and regulator of haematopoiesis. Br J Haematol. 2007;139(2):185–193. 17897294 10.1111/j.1365-2141.2007.06802.x

[31] Geng D, Zheng L, Srivastava R, Asprodites N, Velasco-Gonzalez C, Davila E. When Toll-like receptor and T-cell receptor signals collide: a mechanism for enhanced CD8 T-cell effector function. Blood. 2010;116(18):3494–3504. 10.1182/blood-2010-02-268169 20696947 PMC2981476

[32] Morrison C, Baer MR, Zandberg DP, Kimball A, Davila E. Effects of Toll-like receptor signals in T-cell neoplasms. Future Oncology. 2011;7(2):309–320. 10.2217/fon.10.185 21345147 PMC3463000

[33] Decker EL, Nehmann N, Kampen E, Eibel H, Zipfel PF, Skerka C. Early growth response proteins (EGR) and nuclear factors of activated T cells (NFAT) form heterodimers and regulate proinflammatory cytokine gene expression. Nucleic Acids Res. 2003;31(3):911–921. 12560487 10.1093/nar/gkg186 PMC149206

[34] Miyazaki T, Lemonnier FA. Modulation of thymic selection by expression of an immediate-early gene, early growth response 1 (Egr-1). Journal of Experimental Medicine. 1998;188(4):715–723. 9705953 10.1084/jem.188.4.715 PMC2213358

[35] Basson MA, Wilson TJ, Legname GA, Sarner N, Tomlinson PD, Tybulewicz VLJ, et al Early growth response (Egr)-1 gene induction in the thymus in response to TCR ligation during early steps in positive selection is not required for CD8 lineage commitment. Journal of Immunology. 2000;165(5):2444–2450. 10946269 10.4049/jimmunol.165.5.2444

[36] Perezcastillo A, Pipaon C, Garcia I, Alemany S. NGFI-a gene-expression is necessary for t-lymphocyte proliferation. Journal of Biological Chemistry. 1993;268(26):19445–19450. 8366092

[37] Decker EL, Nehmann N, Kampen E, Eibel H, Zipfel PF, Skerka C. Early growth response proteins (EGR) and nuclear factors of activated T cells (NFAT) form heterodimers and regulate proinflammatory cytokine gene expression. Nucleic acids research. 2003;31(3):911–921. 12560487 10.1093/nar/gkg186 PMC149206

[38] Virolle T, Krones-Herzig A, Baron V, De Gregorio G, Adamson ED, Mercola D. Egr1 promotes growth and survival of prostate cancer cells—Identification of novel Egr1 target genes. Journal of Biological Chemistry. 2003;278(14):11802–11810. 12556466 10.1074/jbc.M210279200

[39] Iniguez MA, Punzon C, Fresno M. Induction of cyclooxygenase-2 on activated T lymphocytes: Regulation of T cell activation by cyclooxygenase-2 inhibitors. Journal of Immunology. 1999;163(1):111–119. 10384106

[40] Kalinski P. Regulation of Immune Responses by Prostaglandin E-2. Journal of Immunology. 2012;188(1):21–28. 10.4049/jimmunol.1101029 PMC3249979 22187483

[41] Bettens F, Kristensen F, Walker C, Schwulera U, Bonnard GD, Deweck AL. Lymphokine Regulation of Activated (G1) Lymphocytes. 2. Glucocorticoid and Anti-Tac-Induced Inhibtion of Human Lymphocyte-T Proliferation. Journal of Immunology. 1984;132(1):261–265. 6606667

[42] Kolenko V, Rayman P, Roy B, Cathcart MK, O'Shea J, Tubbs R, et al Downregulation of JAK3 protein levels in T lymphocytes by prostaglandin E-2 and other cyclic adenosine monophosphate-elevating agents: Impact on interleukin-2 receptor signaling pathway. Blood. 1999;93(7):2308–2318. 10090941

[43] Betz M, Fox BS. Prostaglandin-E2 inhibits production of TH1 lymphokines but not of TH2 lymphokines. Journal of Immunology. 1991;146(1):108–113. 1845802

[44] Baratelli F, Lin Y, Zhu L, Yang SC, Heuze-Vourc'h N, Zeng G, et al Prostaglandin E-2 induces FOXP3 gene expression and T regulatory cell function in human CD4(+) T cells. Journal of Immunology. 2005;175(3):1483–1490. 16034085 10.4049/jimmunol.175.3.1483

[45] Giantin M, Vascellari M, Lopparelli RM, Ariani P, Vercelli A, Morello EM, et al Expression of the aryl hydrocarbon receptor pathway and cyclooxygenase-2 in dog tumors. Research in veterinary science. 2013;94(1):90–99. 10.1016/j.rvsc.2012.07.035 22925934

[46] Xia D, Wang D, Kim S-H, Katoh H, DuBois RN. Prostaglandin E-2 promotes intestinal tumor growth via DNA methylation. Nature Medicine. 2012;18(2):224–226. 10.1038/nm.2608 22270723 PMC3274627

[47] Bakin AV, Curran T. Role of DNA 5-methylcytosine transferase in cell transformation by fos. Science. 1999;283(5400):387–390. 9888853 10.1126/science.283.5400.387

[48] Eferl R, Wagner EF. AP-1: A double-edged sword in tumorigenesis. Nature Reviews Cancer. 2003;3(11):859–868. 14668816 10.1038/nrc1209

[49] Shaulian E, Karin M. AP-1 as a regulator of cell life and death. Nature Cell Biology. 2002;4(5):E131–E136. 11988758 10.1038/ncb0502-e131

[50] Oda E, Ohki R, Murasawa H, Nemoto J, Shibue T, Yamashita T, et al Nora, a BH3-only member of the Bcl-2 family and candidate mediator of p53-induced apoptosis. Science. 2000;288(5468):1053–1058. 10807576 10.1126/science.288.5468.1053

[51] Villunger A, Michalak EM, Coultas L, Mullauer F, Bock G, Ausserlechner MJ, et al p53- and drug-induced apoptotic responses mediated by BH3-only proteins Puma and Noxa. Science. 2003;302(5647):1036–1038. 14500851 10.1126/science.1090072

[52] Burns TF, Fei PW, Scata KA, Dicker DT, El-Deiry WS. Silencing of the novel p53 target gene Snk/Plk2 leads to mitotic catastrophe in paclitaxel (Taxol)-exposed cells. Molecular and Cellular Biology. 2003;23(16):5556–5571. 12897130 10.1128/MCB.23.16.5556-5571.2003 PMC166320

[53] Warnke S, Kemmler S, Hames RS, Tsai HL, Hoffmann-Rohrer U, Fry AM, et al Polo-like kinase-2 is required for centriole duplication in mammalian cells. Current Biology. 2004;14(13):1200–1207. 15242618 10.1016/j.cub.2004.06.059

[54] Ouyang B, Li W, Pan H, Meadows J, Hoffmann I, Dai W. The physical association and phosphorylation of Cdc25C protein phosphatase by Prk. Oncogene. 1999;18(44):6029–6036. 10557092 10.1038/sj.onc.1202983

[55] Yokota I, Sasaki Y, Kashima L, Idogawa M, Tokino T. Identification and characterization of early growth response 2, a zinc-finger transcription factor, as a p53-regulated proapoptotic gene. Int J Oncol. 2010;37(6):1407–1416. 21042708 10.3892/ijo_00000792

[56] Li S, Symonds ALJ, Zhu B, Liu M, Raymond MV, Miao T, et al Early Growth Response Gene-2 (Egr-2) Regulates the Development of B and T Cells. Plos One. 2011;6(4). 10.1371/journal.pone.0018498 PMC3077377 21533228

[57] Harris JE, Bishop KD, Phillips NE, Mordes JP, Greiner DL, Rossini AA, et al Early growth response gene-2, a zinc-finger transcription factor, is required for full induction of clonal anergy in CD4(+) T cells. Journal of Immunology. 2004;173(12):7331–7338. 15585857 10.4049/jimmunol.173.12.7331

[58] Safford M, Collins S, Lutz MA, Allen A, Huang CT, Kowalski J, et al Egr-2 and Egr-3 are negative regulators of T cell activation. Nat Immunol. 2005;6(5):472–480. 15834410 10.1038/ni1193

[59] De Smaele E, Zazzeroni F, Papa S, Nguyen DU, Jin RG, Jones J, et al Induction of gadd45 beta by NF-kappa B downregulates pro-apoptotic JNK signalling. Nature. 2001;414(6861):308–313. 11713530 10.1038/35104560

[60] Li CY, Zhan YQ, Xu CW, Xu WX, Wang SY, Lv J, et al EDAG regulates the proliferation and differentiation of hematopoietic cells and resists cell apoptosis through the activation of nuclear factor-kappa B. Cell Death Differ. 2004;11(12):1299–1308. 15332117 10.1038/sj.cdd.4401490

[61] Li CY, Zhan YQ, Li W, Xu CW, Xu WX, Yu DH, et al Overexpression of a hematopoietic transcriptional regulator EDAG induces myelopoiesis and suppresses lymphopoiesis in transgenic mice. Leukemia. 2007;21(11):2277–2286. 17690693 10.1038/sj.leu.2404901

